# Supplementary material for: SOS-Inducing Drugs Trigger Nucleic Acid Release and Biofilm Formation in Gram-Negative Bacteria
Source: Biomolecules. 2024 Mar 8;14(3):321. doi: 10.3390/biom14030321 (PMC10967838; doi:10.3390/biom14030321)
Supplement: Supplementary file 1 [file biomolecules-14-00321-s001.zip › biomolecules-2872298-supplementary.pdf]

Supplemental Figures S1 and S2.

Supplemental Figure S1.

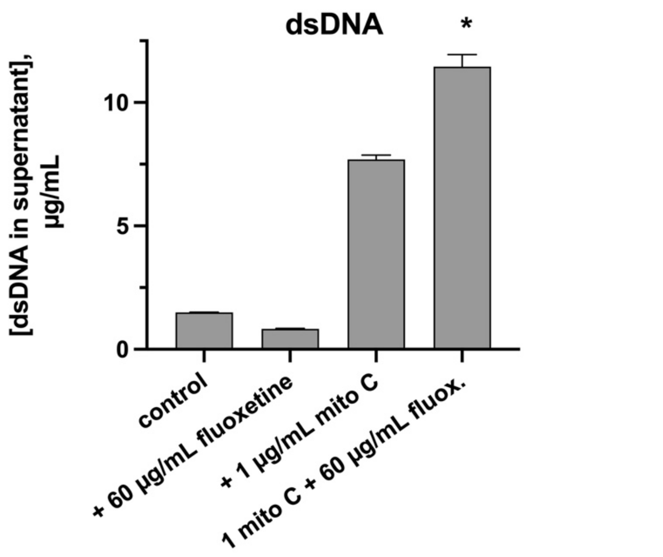

*, significantly greater than mitomycin C alone, $p < 0.01$

Legend to Supplemental Fig. 1. Strain E_clo_Niagara was grown and treated with fluoxetine, mitomycin C, or both, as described in Materials and Methods.

Supplemental Fig. S2

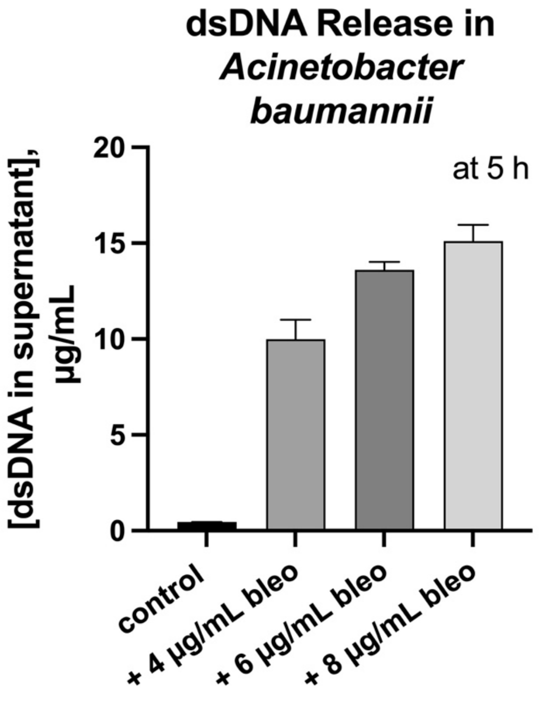

Strain SS_6_13
